# Cardiac Valvular Incompetence Associated With Selective Serotonin Reuptake Inhibitors (SSRIs): A Disproportionality and Risk Factor Analysis From Global Safety Reports

**DOI:** 10.7759/cureus.101519

**Published:** 2026-01-14

**Authors:** Adrian Chin Yan Chan, Yue Han

**Affiliations:** 1 Pharmacology, Bayer Pharmaceuticals, Beijing, CHN; 2 Biostatistics, Cytel, Shanghai, CHN

**Keywords:** 5-ht₂b receptor activation, drug-induced valvulopathy, pharmacovigilance, selective serotonin reuptake inhibitors (ssris), valvular heart disease (vhd)

## Abstract

Background

Selective serotonin reuptake inhibitors (SSRIs) are among the most prescribed antidepressants because of their efficacy and safety. However, new clinical and pharmacological evidence have raised concern about their potential association with valvular heart disease (VHD), which may be mediated by inhibition of the serotonin transporter (SERT) and activation of the 5-hydroxytryptamine receptor 2B (5-HT_2_B receptor).

Methods

In this study, we utilized VigiBase, the World Health Organization's global pharmacovigilance database, to examine potential safety signals related to cardiac valvular incompetence among users of six SSRIs: sertraline, paroxetine, fluoxetine, fluvoxamine, citalopram, and escitalopram. Disproportionality analyses using Information Component (IC) statistics were performed, and complementary logistic regression analyses were used to identify risk factors.

Results

Disproportionality analyses identified significant safety signals for paroxetine and sertraline, particularly for pulmonary and tricuspid valve incompetence. Logistic regression analyses demonstrated that additional cardiac comorbidities and concomitant use of medications associated with serotonin dysregulation and valvopathy were independent risk factors for valvopathy. In the unadjusted analysis, the presence of additional cardiac comorbidities (OR 10.498, 95% CI 5.107-21.579; p < 0.0001) and concomitant use of medications associated with serotonin dysregulation and valvopathy (OR 1.956, 95% CI 1.025-3.732; p = 0.0419) were associated with increased odds of cardiac valvular incompetence. These associations remained significant in the adjusted model for cardiac comorbidities (OR 10.28, 95% CI 5.007-21.109; p < 0.0001) and concomitant medications (OR 2.011, 95% CI 1.055-3.833; p = 0.0337), indicating robustness of findings after covariate adjustment.

Conclusions

The present global pharmacovigilance analysis has revealed safety signals indicating a potential association between three SSRIs (paroxetine, sertraline, and fluoxetine) and cardiac valvular incompetence. Notably, paroxetine and sertraline demonstrated the strongest signal strength. Adjusted logistic regression analysis identified pre-existing cardiac comorbidities and concomitant medications for which there is evidence of serotonin dysregulation and valvopathy as independent predictors of valvopathy. Prospective cohort studies, mechanistic research, and real-world evidence are essential to substantiate these pharmacovigilance signals and to elucidate potential pathophysiological mechanisms.

## Introduction

Selective serotonin reuptake inhibitors (SSRIs) are a class of drugs frequently used in the treatment of depression because of their robust safety, efficacy, and tolerability profiles, making them the drug of choice as a first-line pharmacotherapy [1,2]. Their primary mechanism of action is to occupy the serotonin transporter (SERT) protein located on the presynaptic membrane of serotonergic neurons. This prevents the reuptake of serotonin into presynaptic cells, thus causing an accumulation of serotonin in the extracellular space, which eventually leads to an increased serotonergic transmission [2]. Although these drugs are regarded as "selective," their receptor-binding characteristics are not necessarily selective to SERT [3]. SSRIs exert their primary pharmacological effect through high-affinity inhibition of the SERT, leading to increased synaptic serotonin [4,5].

Drug-induced valvular heart disease (VHD) typically manifests as cardiac-valvular regurgitation and arises from interactions between serotonin (5-HT), the 5-HT transporter (SERT), and the 5-hydroxytryptamine receptor 2B (5-HT_2_B receptor) [6]. One primary mechanism involves direct activation of 5-HT_2_B receptors on the mitral and aortic valves by drugs, which triggers fibrotic transformation through multiple mitogenic pathways. Activation of 5-HT_2_B receptors on valvular interstitial cells triggers several mitogenic and pro-fibrotic pathways involved in drug-induced valvopathy. Consequent to receptor stimulation, Gαq-mediated signaling activates phospholipase C-β (PLC-β), which in turn activates protein kinase C (PKC). PKC acts as a key amplifier, engaging the mitogen-activated protein kinase/extracellular signal-regulated kinase (MAPK/ERK) cascades, promoting fibroblast proliferation and deposition of the extracellular matrix. At the same time, Gαq signalling increases Src phosphorylation, which enhances transforming growth factor-β1. This results in the fibrotic remodeling of valve leaflets. Apart from these G-protein pathways, stimulation of the 5-HT_2_B receptor promotes the phosphorylation of the retinoblastoma protein (pRb), a critical regulator of the cell cycle. This modification inactivates pRb, allowing the valvular fibroblast-like cells to progress through the cell cycle. Cell cycle progression increases cellular proliferation and leads to pathologic thickening of the mitral and aortic valves. These mechanisms all come together to produce the hallmark fibrotic transformation seen in serotonergic drug-associated valvopathy [7,8].

Elevated circulating 5-HT levels constitute another mechanism, as seen in conditions like carcinoid syndrome or in patients taking drugs such as pergolide and cabergoline, with experimental rat models demonstrating structural valve abnormalities following chronic serotonin administration [9]. Impaired SERT function further exacerbates the condition, as this transporter, highly expressed in platelets and pulmonary endothelial cells, normally detoxifies plasma serotonin to protect the heart; reduced SERT activity, such as in knockout mice, decreases 5-HT clearance, resulting in elevated serotonin levels and subsequent valvular damage [10].

Concerns about serotonergic drugs and VHD are not new; for instance, fenfluramine and related anorectic agents were withdrawn from the market after being linked to valvulopathy through 5-HT_2_B receptor activation [11]. This raises the question of whether SSRIs, which share serotonergic activity, might pose a similar risk. In vitro studies have demonstrated that all clinically used SSRIs are 5-HT_2_B receptor agonists and are approximately equipotent towards 5-HT_2_B receptors [12]. Therefore, in patients receiving SSRI therapy, the potential pharmacological mechanism leading to the development of VHD may involve both the blocking of SERTs (especially those located in the in the pulmonary artery endothelium) leading to an elevation of 5-HT levels in the heart and a direct (SERT-independent) stimulation of the 5-HT_2_B receptors located on the aortic and mitral valves, with both SSRI-mediated mechanisms acting synergistically to induce valvopathies [13,14].

A case-control study by De Backer et al. [15] demonstrated a dose-response relationship where higher SSRI/serotonin-norepinephrine reuptake inhibitors (SNRIs) doses and prolonged exposure were associated with increased risk of valvular involvement. However, clinical evidence regarding SSRI-induced VHD remains limited and inconclusive. Moreover, such clinical evidence is primarily derived from case reports, pharmacovigilance alerts, and small observational studies, hence leading to significant uncertainty regarding the magnitude of the risk and the presence of any potential dose-response patterns [16].

## Materials and methods

Study aim

This research aims to assess potential safety signals for SSRI-associated valvular incompetence using global pharmacovigilance data from VigiBase, and to explore patient or drug-related factors that may influence this association.

Study design

The population included patients exposed to six SSRIs (sertraline, fluoxetine, paroxetine, fluvoxamine, citalopram, and escitalopram). The intervention was SSRI exposure, compared against reports of the same drugs without valvular incompetence (non-cases). The primary outcome was cardiac valvular incompetence, defined by Medical Dictionary for Regulatory Activities (MedDRA) terms for aortic, mitral, tricuspid, pulmonary valve incompetence, or unspecified heart valve incompetence. Disproportionality analysis using Information Component (IC) values and logistic regression on individual case safety reports (ICSRs) was employed.

Inclusion and exclusion criteria

Inclusion criteria comprised all reports in VigiBase containing any of the six SSRIs recorded as suspect or interacting drugs. Reports lacking a valid patient, reporter, or suspected drug, as defined under ICH E2B(R2) format, were excluded automatically by the Uppsala Monitoring Centre (UMC)'s data validation process. No further exclusions were applied based on seriousness, age, or region. Reports in all languages were included.

Study data source

This study used data from VigiBase, the World Health Organization (WHO) global pharmacovigilance database, which contains over 19 million reports of suspected adverse drug reactions (ADRs) from more than 130 countries (representing >90% of the world’s population) [17]. These reports, submitted by member nations of the WHO Programme for International Drug Monitoring (PIDM) since 1968, include administrative, patient, medication, and ADR data sourced from healthcare professionals and patients.

Access to VigiBase data was obtained through a formal request to the WHO VigiBase team, which typically grants access to research institutions and regulatory agencies for pharmacovigilance studies. As no patient identifiers were accessed and the analysis involved aggregated, retrospective data, ethical approval was not required for this research.

Design and analysis

VigiBase Disproportionality Analyses

VigiBase provided the ICs for the six commonly prescribed SSRIs (sertraline, fluoxetine, paroxetine, fluvoxamine, citalopram, escitalopram) and adverse events indicative of cardiac valvular incompetence. The reported adverse events, as per MedDRA preferred terms (PTs), include pulmonary valve incompetence, tricuspid valve incompetence, aortic valve incompetence, mitral valve incompetence, and heart valve incompetence.

The disproportionality analysis was performed using IC values, which VigiBase applies as its standard method for signal detection in all pharmacovigilance studies. The ICs reflected the time from each SSRI’s first approval worldwide (i.e., its international birth date) up to 8 June 2024 were included. The specific international birth date for each SSRI is listed in Appendix A. The goal of the analysis was to explore whether specific drug-event combinations were reported more frequently than expected. The ICs are calculated by VigiBase using a Bayesian statistical tool that evaluates the difference between the observed frequency of reports for a particular drug-adverse event pair and the expected frequency, based on the entire dataset. Positive IC values indicate that the observed cases surpass the expected number, while negative IC values suggest underreporting. Statistical significance was determined using the lower bound of the 97.5% CI (IC025), where a positive IC025 (>0) indicates a significant association.

Statistical Analysis

As a complement to the disproportionality analysis, a focused examination of the relationship between all six SSRIs and cardiac valvular incompetence was conducted. ICSRs associated with any of the six SSRIs and reporting any of the adverse event terms indicative of cardiac valvular incompetence were extracted and systematically reviewed. ICSRs included data from the time of each SSRIs' first approval worldwide (i.e., its international birth date) up to 8 June 2024. Data points such as patient demographics (age), concurrently reported events, and drug-related information (including indication, frequency, and dosage) were analyzed. Statistical analyses were conducted using Statistical Analysis System (SAS) software, version 9.4 (SAS Institute Inc., Cary, NC, USA). Adjusted odds ratios (ORs) and corresponding 95% confidence intervals (CIs) were estimated using a logistic regression model, with case/non-case status for cardiac valvular incompetence as the dependent variable.

Case definitions, comorbidity classification, and dose categorization

Case and Non-case Definition

Cases were defined as ICSRs that documented at least one adverse event MedDRA PT indicative of cardiac valvular incompetence (aortic valve incompetence, pulmonary valve incompetence, tricuspid valve incompetence, mitral valve incompetence, or unspecified heart valve incompetence). Non-cases were defined as all other ICSRs for the same six SSRIs that did not report any of these valve-related PTs. This case/non-case structure aligns with standard pharmacovigilance disproportionality methodology.

Definition of Comorbidity Groups

Four comorbidity categories were evaluated as potential confounders: (1) vascular, (2) additional cardiac or valvular, (3) metabolic, and (4) other clinically relevant comorbidities. Each comorbidity category was defined based on co-reported adverse event MedDRA PTs and/or relevant free-text disease terms listed in the indication field of suspect and/or concomitant medications. An ICSR or patient was therefore classified as having a particular category of comorbidity if the case reported at least one adverse event PT or indication term within that corresponding category. Full lists of adverse event PTs and indication terms for each category are provided in Appendix B.

Defined Daily Dose (DDD) Classification

Drug exposure was recoded into two dose categories based on WHO DDD standards: low/standard dose: ≤1 DDD and high dose: >1 DDD. The WHO DDD values [18] for each SSRI included in this analysis are provided in Appendix C.

Use of Concomitant Medications Known to Influence Serotonin and Valvulopathy

Concomitant medications with serotonergic activity or known associations with VHD were identified and classified as potential confounders. These agents may act through SERT inhibition, 5-HT_2_B receptor agonism, or other mechanisms contributing to elevated serotonin levels and fibrotic remodeling of cardiac valves, and are provided in Appendix D.

## Results

Table 1 summarizes demographic distribution and types of heart valve-related adverse events across six SSRIs (sertraline, paroxetine, fluoxetine, escitalopram, citalopram, and fluvoxamine) with a total sample of 1,462 cases. Across all SSRIs, the majority of reports came from patients of unknown age (1088, 69.4%), followed by the ≤17 years group (239, 15.3%), indicating that pediatric and adolescent cases were relatively common. The 18-44-year and 45-64-year groups accounted for smaller proportions (77, 4.9%) and (116, 7.4%), respectively, while only 47 (3.0%) were aged ≥65 years. As the percentage of missing age entries exceeds the threshold of 20%, the independent variable of age has been excluded from the ensuing logistic regression analysis to preserve analytical integrity [19]. The association between age group and SSRI type was statistically significant, as evidenced by a chi-square value of 547.531 with a p-value <0.001, indicating that the distribution of age groups varied significantly across different SSRIs. By gender, the distribution was nearly equal, with males representing 740 (50.6%) and females 718 (49.1%). Despite this apparent balance in overall proportions, the chi-square test demonstrated a statistically significant association between gender and SSRI type (χ^2^=132.718, p<0.001), suggesting that gender distributions differed across individual SSRIs rather than being uniformly distributed. In terms of specific heart valve adverse events, the most frequently reported valvular incompetence event was tricuspid valve incompetence (553, 35.3%), followed by mitral valve incompetence (413, 26.4%), and aortic valve incompetence (323, 20.6%). Pulmonary valve incompetence accounted for 204 (13.0%), while heart valve incompetence (unspecified) events were the least common at 74 (4.7%). The distribution of specific MedDRA PTs across SSRIs was statistically significant (χ^2^=63.857, p<0.001), indicating meaningful differences in the pattern of reported valvular adverse events among the six SSRIs.

**Table 1:** Distribution of age, gender, and reported cardiac valvular events (MedDRA PTs) among ICSRs for six commonly prescribed SSRIs in WHO VigiBase ***p<0.001 MedDRA: Medical Dictionary for Regulatory Activities; PTs: preferred terms; ICSRs: individual case safety reports; SSRIs: selective serotonin reuptake inhibitors

Variable	SSRI						Total	Chi-square (p-value)
	Citalopram (n=28)	Escitalopram (n=54)	Fluoxetine (n=215)	Fluvoxamine (n=10)	Paroxetine (n=601)	Sertraline (n=659)		
Age group (years), n (%)								
<=17	7 (25.0)	5 (9.3)	55 (25.6)	1 (10.0)	90 (15.0)	81 (12.3)	239 (15.3)	547.531 (0.000***)
18-44	0 (0.0)	18 (33.3)	23 (10.7)	6 (60.0)	13 (2.2)	17 (2.6)	77 (4.9)	
45-64	3 (10.7)	11 (20.4)	48 (22.3)	0 (0.0)	27 (4.5)	27 (4.1)	116 (7.4)	
>=65	4 (14.3)	15 (27.8)	10 (4.7)	3 (30.0)	6 (1.0)	9 (1.4)	47 (3.0)	
Unknown	14 (50.0)	5 (9.3)	79 (36.7)	0 (0.0)	465 (77.4)	525 (79.7)	1088 (69.4)	
Gender, n (%)								
Male	8 (33.3)	17 (31.5)	17 (13.3)	7 (70.0)	336 (57.1)	355 (54.0)	740 (50.6)	132.718 (0.000***)
Female	16 (66.7)	37 (68.5)	107 (83.6)	3 (30.0)	252 (42.9)	303 (46.0)	718 (49.1)	
Not known	0 (0.0)	0 (0.0)	4 (3.1)	0 (0.0)	0 (0.0)	0 (0.0)	4 (0.3)	
Event MedDRA PT, n (%)								
Aortic valve incompetence	5 (17.9)	7 (13.0)	61 (28.4)	2 (20.0)	130 (21.6)	118 (17.9)	323 (20.6)	63.857 (0.000***)
Heart valve incompetence	1 (3.6)	4 (7.4)	4 (1.9)	0 (0.0)	30 (5.0)	35 (5.3)	74 (4.7)	
Mitral valve incompetence	11 (39.3)	26 (48.1)	65 (30.2)	5 (50.0)	165 (27.5)	141 (21.4)	413 (26.4)	
Pulmonary valve incompetence	3 (10.7)	4 (7.4)	24 (11.2)	1 (10.0)	56 (9.3)	116 (17.6)	204 (13.0)	
Tricuspid valve incompetence	8 (28.6)	13 (24.1)	61 (28.4)	2 (20.0)	220 (36.6)	249 (37.8)	553 (35.3)	

Table 2 shows the results of disproportionality analysis as quantified by IC values, where three of the six SSRIs (paroxetine, sertraline, and fluoxetine) demonstrated safety signals for events indicative of cardiac valvulopathy. Table 3 shows the unadjusted and adjusted logistic regression analysis performed using the backward stepwise selection method. In the unadjusted model, patients with additional cardiac comorbidities (OR 10.498, 95% CI 5.107-21.579, p<0.0001), and patients receiving concomitant medications for which there is evidence of serotonin dysregulation and valvopathy (OR 1.956, 95% CI 1.025-3.732, p=0.0419) were associated with higher odds of cardiac valvular incompetence, while the other variables showed no significant associations.

**Table 2:** Results of disproportionality analysis of SSRI-associated heart valve incompetence events, ranked by their IC values MedDRA: Medical Dictionary for Regulatory Activities; PTs: preferred terms; IC: Information Component; SSRIs: selective serotonin reuptake inhibitors

SSRI name	Event MedDRA PT	Cases Reported	Cases Expected	IC	IC025	IC975
Paroxetine	Pulmonary valve incompetence	43	2.205	4.007	3.544	4.406
Sertraline	Pulmonary valve incompetence	40	2.574	3.720	3.238	4.132
Paroxetine	Tricuspid valve incompetence	162	12.693	3.623	3.392	3.836
Paroxetine	Aortic valve incompetence	99	12.679	2.916	2.618	3.186
Sertraline	Tricuspid valve incompetence	108	14.820	2.824	2.539	3.083
Fluoxetine	Pulmonary valve incompetence	20	2.413	2.815	2.114	3.378
Paroxetine	Mitral valve incompetence	117	23.173	2.311	2.038	2.561
Paroxetine	Heart valve incompetence	22	4.965	2.042	1.376	2.581
Sertraline	Aortic valve incompetence	58	14.804	1.935	1.539	2.282
Fluoxetine	Aortic valve incompetence	48	13.875	1.754	1.317	2.133
Fluoxetine	Tricuspid valve incompetence	46	13.890	1.692	1.245	2.079
Sertraline	Heart valve incompetence	17	5.797	1.475	0.708	2.079
Escitalopram	Pulmonary valve incompetence	4	1.270	1.346	-0.390	2.426
Sertraline	Mitral valve incompetence	67	27.056	1.293	0.926	1.617
Fluoxetine	Mitral valve incompetence	54	25.359	1.076	0.665	1.434
Citalopram	Pulmonary valve incompetence	3	1.201	1.041	-1.009	2.235
Fluvoxamine	Pulmonary valve incompetence	1	0.322	0.867	-2.931	2.507
Escitalopram	Tricuspid valve incompetence	12	7.311	0.678	-0.252	1.380
Fluvoxamine	Mitral valve incompetence	5	3.388	0.500	-1.027	1.495
Escitalopram	Heart valve incompetence	4	2.860	0.422	-1.315	1.501
Escitalopram	Mitral valve incompetence	17	13.347	0.338	-0.429	0.942
Citalopram	Tricuspid valve incompetence	8	6.916	0.197	-0.971	1.025
Fluvoxamine	Aortic valve incompetence	2	1.854	0.087	-2.501	1.447
Fluvoxamine	Tricuspid valve incompetence	2	1.856	0.086	-2.503	1.446
Escitalopram	Aortic valve incompetence	7	7.303	-0.057	-1.317	0.817
Fluoxetine	Heart valve incompetence	4	5.434	-0.399	-2.136	0.681
Citalopram	Aortic valve incompetence	5	6.908	-0.430	-1.957	0.565
Citalopram	Mitral valve incompetence	9	12.626	-0.466	-1.560	0.324
Citalopram	Heart valve incompetence	1	2.705	-1.096	-4.893	0.544

**Table 3:** Logistic regression of risk factors for SSRI-associated valvulopathy dependent variable: 0: non-case, 1: case, **p<0.0001, *p<0.05 ^1^ Backwards stepwise binary logistic regression is used in the analysis. Removal testing was based on the probability of the Wald statistic (p-value of entry 0.05 and removal 0.10). Four variables were removed from the model: other significant comorbidities/confounders, vascular comorbidities/confounders, metabolic comorbidities/confounders, and DDD. The model fit well as measured by the Hosmer and Lemeshow test (model X^2^=4.6418, DF=4, P=0.3261). SSRI: selective serotonin reuptake inhibitor; DDD: defined daily dose

Factor	Logistic regression (unadjusted)				Logistic regression (adjusted^1^)			
	OR	CI	Wald chi-square	P-value	OR	CI	Wald chi-square	P-value
Vascular comorbidities/confounders	<0.001	<0.001->999.999	0.0014	0.9701	-	-	-	-
Additional cardiac comorbidities/confounders	10.498	5.107-21.579	40.9072	<0.0001**	10.28	5.007-21.109	40.2974	<0.0001**
Metabolic comorbidities/confounders	1.677	0.232-12.118	0.2625	0.6084	-	-	-	-
Other significant comorbidities/confounders	<0.001	<0.001->999.999	0.0000	0.9947	-	-	-	-
Medications for which there is evidence of serotonin dysregulation and valvopathy	1.956	1.025-3.732	4.1410	0.0419*	2.011	1.055-3.833	4.5081	0.0337*
DDD (high vs. low)	1.369	0.938-1.997	2.6515	0.1035	-	-	-	-

In the adjusted model, patients with additional cardiac comorbidities (OR 10.28, 95% CI 5.007-21.109, p<0.0001) and patients receiving concomitant medications for which there is evidence of serotonin dysregulation and valvopathy (OR 2.011, 95% CI 1.055-3.833, p=0.0337) remain as the two variables associated with higher odds of cardiac valvular incompetence. Results were robust to adjustment for covariates, with findings remaining statistically significant.

## Discussion

Summary of principal findings

This pharmacovigilance investigation used the VigiBase database from the WHO to systematically assess the association between SSRIs and cardiac valvular incompetence. The findings indicated a disproportionality in reporting VHD, with paroxetine and sertraline showing the strongest signals and fluoxetine to a lesser extent, confirmed by IC values (paroxetine: IC=4.007 for pulmonary valve incompetence and IC=3.623 for tricuspid valve incompetence; sertraline: IC=3.720, IC=2.824). This observation correlates with their higher binding affinity to the SERT, as shown in Appendix E, supporting the hypothesis that SSRI-induced valvular incompetence may be mediated by SERT blockade, particularly in pulmonary artery endothelium, leading to elevated circulating serotonin and subsequent valvular remodelling. Adjusted logistic regression analysis identified pre-existing cardiac comorbidities and concomitant medications for which there is evidence of serotonin dysregulation and valvopathy as independent predictors of valvopathy. In contrast, SSRIs such as escitalopram, citalopram, and fluvoxamine presented little or no signal in assessing risk of valvopathy, indicating drug-specific risk [20].

Clinical implications

This analysis has revealed safety signals indicating a potential association between the SSRIs of paroxetine, sertraline, and fluoxetine and cardiac valvular incompetence. However, adjusted logistic regression analysis reveals that these signals appear to be confounded by pre-existing cardiac conditions and by concomitant medications for which there is evidence of serotonin dysregulation and valvopathy. Pharmacological insights suggest that SSRIs may contribute to valvular damage via agonism of 5-HT_2_B receptors and inhibition of the SERT, leading to elevated circulating serotonin levels. Further cohort studies and clinical trials are needed to validate the potential safety signal of SSRI use and its association with cardiac valvular incompetence events [21,22].

Strengths and limitations of the study

The main strength of this study is its use of VigiBase, an international database of reports of ADR, which has data from over 130 countries, and represents more than 19 million ICSRs [17]. This enhances the generalizability of the findings. The use of Bayesian disproportionality analysis and a multivariable logistic regression analysis contributes to the rigor of the methods used and allows for signal detection in multiple populations [23]. Incorporating WHO DDD enables exploration of dose-related effects, which is clinically relevant.

Spontaneous reporting can bias reporting, including underreporting as well as stimulated reporting following regulatory alerts. The high number of cases that lacked age data also limits any demographic comparison. The study does not have clinical validation (e.g., echocardiographic confirmation), nor is there an indication of a temporal relationship between starting SSRI antidepressants and valvopathy, which limits causal inference. Further, confounding by indication, wherein the diagnosis of depression may increase cardiovascular risk, may also bias results [24].

Future directions

Future research should aim to better understand SSRI-related valvulopathy through prospective cohort studies with echocardiographic assessments to establish valve structural alterations. Pathway mechanistic studies of 5-HT_2_B receptor activation, SERT inhibition, and fibrotic signalling are needed to elucidate the pathophysiology [25]. Genetic studies, specifically SERT polymorphisms, can potentially identify higher-risk individuals. Comparative safety analyses across antidepressant classes may establish if this risk is specific to SSRIs or shared among other serotonergic medications. Real-world evidence drawn from electronic health records and imaging databases should also be used to investigate dose thresholds, treatment duration, and patient-level risk factors.

## Conclusions

This global pharmacovigilance analysis has revealed safety signals indicating a potential association between three SSRIs (paroxetine, sertraline, and fluoxetine) and cardiac valvular incompetence. Notably, paroxetine and sertraline demonstrated the strongest signal strength. The signal, however, appears to be confounded by pre-existing cardiac comorbidities and by concomitant medications for which there is evidence of serotonin dysregulation and valvopathies according to our adjusted logistic regression model. Additionally, our adjusted logistic regression analysis did not demonstrate a statistically significant dose-response relationship for cardiac valvular incompetence in patients receiving high doses (>1 DDD) of SSRI when compared to patients receiving low/standard dose SSRI.

It is imperative to recognize the methodological constraints inherent to spontaneous reporting systems. These include reporting bias, confounding by indication, and the absence of echocardiographic validation. Prospective cohort studies, mechanistic research, and real-world evidence are essential to substantiate these pharmacovigilance signals and to elucidate potential pathophysiological mechanisms. Such evidence will be critical in shaping regulatory frameworks, clinical guidelines, and prescribing practices.

## Appendices

Appendix A 

**Table 4:** International birth dates of selective serotonin reuptake inhibitors (SSRIs)

SSRI	International Birth Date (First Global Approval)	Country of First Approval
Fluoxetine	December 29, 1987	United States (FDA)
Fluvoxamine	October 1983	Switzerland (Swissmedic)
Paroxetine	January 1991	United Kingdom (MHRA)
Sertraline	December 30, 1991	United States (FDA)
Citalopram	1989	Denmark (Danish Medicines Agency)
Escitalopram	2001	Denmark (Danish Medicines Agency)

Appendix B 

**Table 5:** Comorbidity definitions PT: preferred terms; HLT: high-level terms

Comorbidity Category	Co-reported Event PT/HLTs	Indication Text Terms
Hypertensive Comorbidities	Autonomic dysreflexia, Catecholamine crisis, Cushingoid, Dialysis induced hypertension, Diastolic hypertension, Essential hypertension, Hyperadrenocorticism, Hypertension, Hypertension neonatal, Labile hypertension, Maternal hypertension affecting foetus, Metabolic syndrome, Neurogenic hypertension, Nocturnal hypertension, Orthostatic hypertension, Paradoxical pressor response, Paroxysmal autonomic instability with dystonia, Postoperative hypertension, Prehypertension, Procedural hypertension, Secondary hypertension, Supine hypertension, Syndrome Z, Systolic hypertension, Venous hypertension, White coat hypertension, Withdrawal hypertension, Aortic arteriosclerosis, Acute aortic syndrome, Aortic aneurysm, Aortic aneurysm rupture, Aortic aneurysm syphilitic, Aortic dissection, Aortic dissection rupture, Aortic intramural haematoma, Distal stent-induced new entry, False lumen dilatation of aortic dissection, Penetrating aortic ulcer	Aortic aneurysm, Aortic arteriosclerosis, Coronary atherosclerosis, Atherosclerosis, Atherothrombosis prophylaxis, Atherosclerosis prophylaxis, Atherosclerotic cardiovascular disease, Cerebral atherosclerosis, Coronary atheroma, Atherosclerosis of arteries of the extremities, Coronary artery atheroma, Vascular atheroma, Hypertension (and all related subtypes such as essential, arterial, pulmonary, benign, malignant, systolic, secondary, etc.), Angina pectoris with hypertension, Uncontrolled hypertension, Hypertension not adequately controlled, etc.
Cardiac/Cardiac Valvular Comorbidities	Abiotrophia defectiva endocarditis, Acute endocarditis, Rheumatic heart disease, Pulmonary oedema, Heart failure (acute/chronic/left/right/congestive), Cardiomyopathy (hypertrophic, dilated, restrictive, toxic, viral, ischaemic, etc.), Pulmonary hypertension (primary, secondary, venous, neonatal, etc.), Arrhythmia, Atrial fibrillation, Cardiac valve disease (aortic, mitral, tricuspid, pulmonary — stenosis, sclerosis, incompetence, thickening, etc.), Congenital valve disorders, Prosthetic valve complications, Cardiogenic shock, Cor pulmonale, Cardiorenal syndrome, Cardiac cirrhosis	Cardiac operation, Rheumatic heart disease, Endocarditis (and all subtypes), Cardiac valve disease, Aortic/mitral/tricuspid valve replacement or repair, Heart valve disorders, Valvuloplasty, Cardiomyopathy (all types), Heart failure (acute/chronic/preserved/reduced EF, etc.), Cardiac failure (all forms), Atrial fibrillation (all types), Heart valve prosthesis presence, Transcatheter aortic valve replacement, Ischemic cardiomyopathy, Hypertensive cardiomyopathy, etc.
Metabolic Comorbidities	Obesity, Central obesity, Overweight, Hypercholesterolaemia (all subtypes), Hyperlipidaemia (Type I–V, mixed, familial, acquired), Hypertriglyceridaemia, Diabetes mellitus (Type 1, 2, gestational, steroid-induced, latent autoimmune, neonatal, etc.), Achard Thiers syndrome, Lipodystrophy, Cystic fibrosis-related diabetes, Insulin resistance, Increased/decreased insulin requirement, Diabetes complicating pregnancy	Diabetes mellitus (all forms and related complications), Type 1 and Type 2 diabetes, Gestational diabetes, Obesity (including morbid, unspecified, post-surgery), Tobacco user/ex-user, Hypercholesterolaemia (familial, primary, pure, high cholesterol), Diabetic complications (neuropathy, nephropathy, foot ulcer, retinopathy), Insulin-dependent diabetes mellitus, Steroid-induced diabetes, Diabetic peripheral neuropathic pain, etc.
Other Comorbidities	Radiotherapy to lung/breast, Carcinoid syndrome, Electronic cigarette user, Ex-tobacco user, Passive smoking, Chronic kidney disease (CKD), End-stage renal disease (ESRD), Tobacco use/exposure terms	Chronic kidney disease (Stage 1–5), End-stage renal disease, Smoking cessation therapy, Ex-smoker, Smoker, Carcinoid syndrome, Heavy smoker, Cessation of smoking

Appendix C 

**Table 6:** WHO DDDs DDD: defined daily dose

ATC Code	Name	DDD	U	Adm.R	Note
N06AB03	fluoxetine	20	mg	O	
N06AB04	citalopram	20	mg	O	
		20	mg	P	
N06AB05	paroxetine	20	mg	O	
N06AB06	sertraline	50	mg	O	
N06AB08	fluvoxamine	0.1	g	O	
N06AB10	escitalopram	10	mg	O	

Appendix D 

**Table 7:** Concomitant medications

Category	Examples
Analgesics and Antidepressants	Tramadol, Trazodone, Amitriptyline, Melitracen, Flupentixol
Herbal Products	*Hypericum perforatum* (St. John’s Wort)
Psychostimulants	Methylphenidate
Anorectic Agents	Fenfluramine, Dexfenfluramine, Benfluorex, Sibutramine, Mazindol
Ergot Derivatives	Pergolide, Cabergoline, Methysergide, Ergotamine
Serotonin-Norepinephrine Reuptake Inhibitors (SNRIs)	Desvenlafaxine, Duloxetine, Levomilnacipran, Milnacipran, Venlafaxine
Monoamine Oxidase Inhibitors (MAOIs)	Phenelzine, Tranylcypromine, Selegiline, Isocarboxazid, Moclobemide
Other Agents	Nefazodone, Nefopam
Metabolic Agents	Hyperlipidemia-Related Drugs

Appendix E 

**Table 8:** Binding affinity values of SSRIs against the SERT SSRIs: selective serotonin reuptake inhibitors; SERT: serotonin transporter

SSRI	SERT Ki (nmol/L)
Paroxetine	0.1
Sertraline	0.26
Fluoxetine	1.1
Escitalopram	1.1
Citalopram	1.6
Fluvoxamine	2.3
